# Editorial: Tips for early career researchers (ECRs) in searching the literature and in academic publishing

**DOI:** 10.1017/S003118202510142X

**Published:** 2026-01

**Authors:** John Ellis, J. Russell Stothard

**Affiliations:** 1 School of Life Sciences, University of Technology Sydney, Ultimo, NSW, Australia; 2 Department of Tropical Disease Biology, Liverpool School of Tropical Medicine, Liverpool, UK

**Keywords:** semiotics, social action, social interaction

## Abstract

**Figure:**
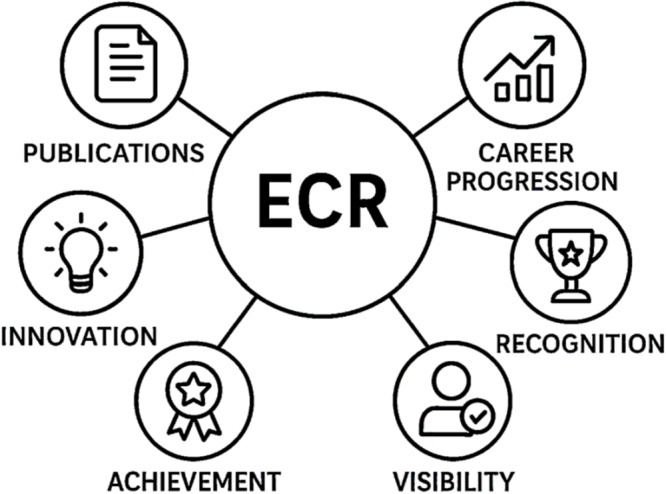

In this 2026 editorial of *Parasitology*, we highlight early career researchers (ECRs) both as our readers and, more often than not, as our authors too. To help them advance in their career and hopefully transition into secure employment, we provide some tips about searching the literature and our perspectives on the role and importance of academic publishing.

## Who and what is an early career researcher?

ECRs are broadly defined by institutions and funders as individuals within 7–10 years of completing a PhD, though a narrower definition, often researchers under 35 who are pursuing or have completed a doctorate without a tenured position, captures the growing cohort of highly skilled yet precariously employed scholars who make up the largest share of the global academic workforce. Valued for their openness, creativity and transparency, ECRs play central roles in publishing, reviewing, project leadership and other core research activities, positioning them as key drivers of shifts in scholarly communication and the future of research systems. However, definitions of ECRs vary considerably, relying on factors such as career age, job title, academic experience, years since PhD or first publication, publication outputs and allowances for career interruptions, underscoring the complexity and diversity of ECR trajectories (Frandsen and Nicolaisen 2024).

## Publish or perish

**Figure 1.**
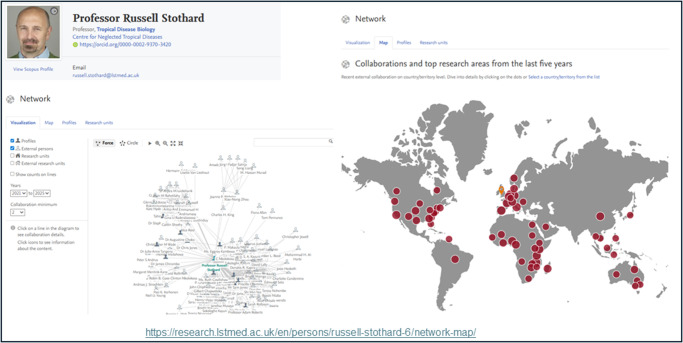
Example webpage containing information to promote and publicize the international connections and societal impacts of Professor Stothard.

‘*Publish or perish*’ captures the intense pressure on academics, especially ECRs, to continually publish in high-impact journals to secure jobs, funding and tenure. This culture often prioritizes quantity and prestige over the quality or societal value of research, pushing researchers to focus on producing citable work rather than engaging in open science, collaboration or public communication. While some academics critique the system, many comply with it out of necessity, knowing their careers depend on peer-reviewed publication metrics. The result is a highly competitive environment where strategic publishing often outweighs innovation or broader impact (Golhasany and Harvey 2022). It is no coincidence that many Higher Education Institutions (HEIs) are populating their staff webpages with information in an attempt to promote and publicize their international connections and societal impacts (Figure 1).

## Benefits of publishing during a PhD

Publishing during a PhD offers numerous benefits for students, supervisors, institutions and the broader research community. It enhances students’ motivation, provides timely feedback, improves writing and research skills and helps build a strong academic profile for future employment. Publishing early also reduces post-PhD publishing pressure and ensures the timely dissemination of research. Supervisors benefit through co-authorship and quicker completions, while institutions gain more publications and better returns on investment. However, there are challenges: students may face anxiety, rejection and increased workload, and the lengthy publication process can delay results. Despite these drawbacks, publishing during candidature supports academic development and visibility, even if a PhD is not completed (Pickering and Byrne 2014). It is also important to consider the ‘soft’ literature around a publication, for example, writing an online digest article to help disseminate your findings to a broader audience. The latter is an essential component of effective scientific writing and communication skills.

## How and where do ECRs publish?

As current international metrics stand, ECRs are productive, averaging 10 journal articles and 6 conference papers, often working within institutional authorship policies that vary by country and discipline. They typically aim to publish in high-impact journals to build a reputation and secure tenure, even though many recognize the value of open access and data sharing. Of note, ECRs have limited autonomy, often constrained by group dynamics, institutional expectations, and the need to conform to traditional publishing norms. While they influence publication decisions and sometimes have a strategy, it usually centres on maximizing output in prestigious journals. Non-traditional dissemination methods like social media are underused due to time constraints and a lack of recognition. Though many ECRs acknowledge flaws in the current system, most accept it (Nicholas et al., 2017).

## Tips on conducting a literature review

### Traditional database search with a strategic focus

For ECRs, traditional literature searches through databases like PubMed, Scopus, Web of Science, Google Scholar and Dimensions remain foundational, particularly in accessing ‘hard’ literature databases that are curated by various international agencies such as Clarivate, amongst others, and responsible for journal impact factor calculations and annual metrics. The key to maximizing search algorithms and effective approaches is developing a clear set of strategic keywords that align closely with the research question. Using Boolean operators (AND, OR, NOT) allows for more precise searches and reduces irrelevant results. It is also important to refine search results by applying filters such as publication date, study type, article type and subject area (Ellis et al., 2020).

To further streamline the process, ECRs can use citation chasing, which involves reviewing the references of relevant articles to identify additional key sources. This can lead to additional snowballing where further cross-checking of the source of information for its reliability and accuracy is possible. This traditional method, when combined with an organized system of managing references (e.g. Zotero, Mendeley or EndNote), ensures a comprehensive, yet focused, literature review with an associated historical timeline of events or evolution of ideas. The latter is particularly beneficial if you are either supporting or refuting scientific norms, placing your ideas within a better context and evidencing novelty.

### Tools for visualizing research trends

For a more in-depth understanding of the research landscape, ECRs can benefit from tools like SciMAT (https://sci2s.ugr.es/scimat/) and VOSviewer (https://www.vosviewer.com/). These software applications allow researchers to visualize citation networks, keyword trends, and the evolution of topics within a field (Ellis et al., 2020). By mapping relationships between key papers, authors and concepts, ECRs can identify leading researchers, core themes and emerging areas. SciMAT, for instance, uses a strategic mapping approach to identify gaps in research, providing ECRs with insights into underexplored topics (Cobo et al., 2012). VOSviewer creates network maps based on co-citation and bibliographic data, offering an easy-to-interpret visual representation of research patterns (Van Eck and Waltman 2010). These tools enable ECRs to conduct more targeted searches and ensure their literature review is not just comprehensive but also strategically aligned with current research directions.

### New tools for focused exploration

Emerging tools like ConnectedPapers (https://www.connectedpapers.com/) and ResearchRabbit (https://www.researchrabbit.ai) offer unique ways to explore literature beyond traditional search methods. These platforms enable researchers to visualize connections between academic papers, creating a ‘graph’ of related works that can help uncover hidden connections between key studies. ConnectedPapers builds a tree of related papers starting from a single seminal work, while ResearchRabbit allows users to track specific topics, authors or papers, sending alerts when new, relevant publications are released. These tools are particularly useful for ECRs seeking to understand the broader context of their research and identify pivotal works that may not appear in typical keyword-based searches. By leveraging these tools, ECRs can refine their searches and build a more nuanced, forward-thinking literature review that is aligned with the latest developments in their field.

## Manuscript preparation and submission

We always recommend that ECRs (and all other authors) follow the guidelines (often called Instructions for Authors or something similar) provided by the Journal they intend to publish in. *Parasitology’s* guidelines can be found here: https://www.cambridge.org/core/journals/parasitology/information/author-instructions.

Submission of manuscripts is through Manuscript Central ScholarOne (https://mc.manuscriptcentral.com/par); first-time users can register for an online account. Queries about manuscript preparation or submission can be made by email to the *Parasitology* administrative office on parasitology@cambridge.org.

### Early career researchers and change

Change is a constant in academic life including ECRs’ scholarly practices. However, traditional systems of publishing, metrics and assessment remain deeply entrenched, limiting faster progress. ECRs often shift their viewpoints, suppressing their values of openness and transparency to align with conventional success metrics needed for job security (Nicholas et al., 2019). However, open access publishing is one area in which this journal has evolved in recent years (Stothard and Ellis 2022).

### Using large language models to improve research writing

Typically, with the rise of Artificial Intelligence (AI) online algorithms, like all researchers worldwide, ECRs can use large language models (LLMs) such as ChatGPT to help polish and clarify their writing (Kim et al., 2023). These tools are especially useful for improving grammar, simplifying complex sentences and ensuring that the overall tone and structure of a research paper are appropriate for the intended audience. Non-native English speakers may find LLMs helpful for refining word choice and enhancing readability. LLMs can also assist in organizing sections more clearly or rephrasing text to meet journal formatting requirements. However, while these tools are powerful for editing and style, they should not be used to generate original scientific ideas, data or interpretations. This is sometimes difficult to objectively recognize, as the LLMs often raise new issues that were not previously identified. The researcher must remain the primary author of any publication and take full responsibility for all content. Indeed, overuse of LLMs is a trap which the less aware should not fall into: if, for example, the ECR has to defend their work by oral presentation, job interview or viva, academic and intellectual weakness are quickly revealed.

As with the application of all new technology, the use of LLMs in academic writing raises important ethical questions (Haltaufderheide and Ranisch 2024). Researchers should be transparent about using these tools, especially when they play a significant role in editing the manuscript. Many journals, *Parasitology* included, now encourage or require authors to disclose LLM use in the acknowledgements. Since LLMs cannot ‘think’, understand context or verify facts (*though they may appear to do so*) human authors must carefully review and validate any text they produce. Importantly, LLMs must never be listed as co-authors, as they cannot take responsibility for the work. To avoid privacy risks, researchers should not share sensitive or unpublished data with online LLMs. Responsible use means using these tools to support, not replace, human judgment and scientific integrity (Sen Gupta 2025).

Some academic journals have strict policies that prohibit the use of LLMs, such as ChatGPT, in any part of the manuscript preparation process (Liang et al., 2024). These restrictions are often based on concerns about authorship, accuracy and accountability. Since LLMs cannot verify facts, understand context or take responsibility for content, journals worry that their use may compromise the integrity of the research.

Additionally, there is concern that LLM-generated text might include unintentional plagiarism or factual errors that are difficult to detect. Journals with these policies typically state that all writing and editing must be done solely by the named authors or professional editors who can be held accountable. Submitting a manuscript that has been edited or written with the help of an LLM may violate submission guidelines and could lead to rejection or retraction. Researchers should always check the editorial policies of their target journals before using LLMs, as requirements vary widely and are evolving quickly. When in doubt, it is safest to avoid LLM use or to seek written clarification from the journal.

The use of generative AI in Cambridge Journals is covered by an AI in research publishing policy (https://www.cambridge.org/core/journals/parasitology/information/author-instructions/preparing-your-materials#useofai), which outlines acceptable use. Editors of *Parasitology* also check the originality of manuscripts submitted to the Journal using iThenticate.

## The use of preprint servers

Preprint servers like arXiv, bioRxiv and medRxiv offer ECRs a valuable way to share their work quickly and openly before formal peer review. Posting a preprint can help increase visibility, establish priority for new findings and demonstrate research activity when applying for jobs or grants. Preprints are freely accessible, which means your work can reach a wider audience and potentially attract useful feedback or new collaborations.

There are, however, important considerations that need careful deliberation and personal reflection before being used. Foremost, preprints are not peer-reviewed, so the responsibility for accuracy and clarity rests entirely with the authors. In some fields, especially medicine or public health, sharing unreviewed results can raise ethical concerns if findings are controversial or could be misinterpreted (Ravinetto et al., 2021). While most journals now accept submissions that have appeared as preprints, a few still do not, so it is important to check a journal’s policy before posting. Overall, when used thoughtfully, preprints can be a powerful tool for ECRs to increase the reach and impact of their work, build their academic profile and take part in the culture of open science.

Cambridge University Press policy (https://www.cambridge.org/core/open-research/preprint-policy) permits authors to share pre-submission versions of their manuscripts on preprint servers at any time and under any license. Authors are encouraged to cite the preprint in their submitted manuscript and to link the final published ‘Version of Record’ to the preprint once it is published. It is also recommended to update the preprint to link to the final published version.

## Publishing and intellectual property protection

For ECRs, publishing research early can lead to potential issues with intellectual property (IP) protection. When research findings are publicly disclosed in journals, conferences or online repositories, they may be considered ‘prior art’, which can limit opportunities for patenting or securing other IP rights later on (Poddar and Rao 2024). This is particularly problematic for ECRs working on novel ideas or technologies that could have commercial value. While open access and rapid dissemination are important, ECRs must be cautious about publishing too early, as it can undermine their ability to patent or protect innovative aspects of their work.

Some journals and funding bodies may also require specific confidentiality agreements or embargoes before certain aspects of research are made public. ECRs should consult with their institution’s technology transfer office or legal advisors before making decisions about when and where to publish, especially if their work has commercial or patent potential. Early disclosure without proper IP safeguards can significantly hinder the ability to commercialize research findings or secure funding for future developments, making careful planning essential to balancing the desire for publication with the need for IP protection.

## ECRs and peer review

ECRs are highly active in peer review, with most conducting reviews and responding to reviewer comments, showing they are junior in title but not in contribution. While generally trusted, peer review is seen by ECRs as essential yet in need of reform, with improvements like double-blind reviews, better reviewer selection and reviewer recognition suggested. Humanities ECRs are less engaged due to different publishing norms. Over half consider peer review type important when selecting what to read. AI is expected to enhance peer review by detecting plagiarism, improving speed and matching reviewers, though few believe it will fully replace humans (Nicholas et al., 2025).

ECRs are highly active and capable participants in peer review, often contributing significantly, even ghost-writing reviews for senior colleagues, despite limited recognition. Studies show no evidence that ECRs perform worse than senior scholars; in fact, their reviews are often more detailed and improvement-focused. ECRs tend to accept review invitations more readily and invest more effort, viewing peer review as a valuable career opportunity (Rodríguez‐Bravo et al., 2017). However, they may lack confidence and feel intimidated by the task. ECRs are also more motivated by rewards and personal gains, unlike senior scholars who see peer review as a duty.

Notably, while Chinese scholars publish extensively, their participation in international peer review is low, despite high acceptance rates when invited. Differences in how ECRs and senior colleagues view the purpose of peer review, improvement versus gatekeeping, highlight the need to better support and integrate ECRs into the system, especially in regions like China, where potential remains underutilized (Wang et al., 2022).

## Recognising the value of social media

Social media offers ECRs a powerful platform for networking, collaboration and career development (Chatzea et al., 2022). By engaging with academic communities on platforms like X (formerly known as Twitter), LinkedIn and ResearchGate, ECRs can build connections with peers, mentors and established researchers in their field. These connections may lead to collaborative opportunities and invitations to present at conferences or co-author papers, which can significantly enhance an ECR’s visibility and career trajectory. Social media also enables ECRs to share their research findings widely, increasing the impact and recognition of their work.

Raised visibility, responsibly gained, can attract attention from funding bodies, institutions and potential collaborators, all of which are essential for advancing in an academic career. Furthermore, platforms like X (Twitter), BlueSky and LinkedIn allow researchers to join conversations around current trends and key topics, helping them stay informed, refine their research and contribute to ongoing debates (Ellis and Reichel 2023). Additionally, social media can serve as an informal space for sharing career advice, discussing challenges and seeking guidance from others who have navigated similar paths. This is particularly helpful when meeting other researchers for the first time at conferences and helping to establish collaborative networks built upon mutual respect and trust.

For ECRs, being active on social media is not just about promoting their own work, but also about establishing a professional online presence that reflects their expertise, values and aspirations, which can be crucial in a competitive academic landscape.

*Parasitology* has dedicated social media editors (https://www.cambridge.org/core/journals/parasitology/information/about-this-journal/editorial-board), whose role is to liaise with and disseminate journal content within the parasitology research community.

## Outlook

We hope this editorial has provided some new ideas and perspectives on establishing a long- term career in parasitology. Looking ahead, Cambridge University Press & Assessment will support a 1-day public seminar during the annual Cambridge Science Festival in Spring 2026 and feature the latest research on ancient parasites and human history. In Autumn 2026, there will be a free online webinar to expand upon this editorial and set to engage with ECRs across the world. Please keep up-to-date with the journal’s website and social media accounts.

## Supporting information

Ellis and Stothard supplementary material Ellis and Stothard supplementary material

## Supplementary material

The supplementary material for this article can be found at https://doi.org/10.1017/S003118202510142X.
