# Editorial: Tips for early career researchers (ECRs) in searching the literature and in academic publishing – ADDENDUM

**DOI:** 10.1017/S0031182026101838

**Published:** 2026-01

**Authors:** John Ellis, J. Russell Stothard

The authors would like to add the following text to the Acknowledgements section:

The Editorial Board would like to thank everyone who kindly reviewed papers for *Parasitology* in 2025. The full list of reviewers can be found in the supplementary material attached to this Editorial.

**Supplementary material.** The Supplementary Material for this paper can be found at DOI: https://doi.org/10.1017/S003118202510142X.
