# Supplementary material for: Editorial: Tips for early career researchers (ECRs) in searching the literature and in academic publishing
Source: Parasitology. 2026 Jan 21;153(1):1–5. doi: 10.1017/S003118202510142X (PMC13215757; doi:10.1017/S003118202510142X)
Supplement: Ellis and Stothard supplementary material [file S003118202510142Xsup001.docx]

**List of Reviewers, 2025**

*Parasitology* would like to thank the following people, who kindly provided reviews for the journal in 2025:

Gema Alvarez-Garcia, Complutense University of Madrid, Spain

Beatriz Andrade, UERJ, Brazil

Daniela de Angeli Dutra, University of Otago, Brazil

Dmitry A. Apanaskevich, Georgia Southern University, United States

Julio C. Aguiar, University of Campinas, Brazil

Lorena Ailán-Choke, Universidad Nacional de Salta, Argentina

Gema Alama-Bermejo, Biology Centre Czech Academy of Sciences Institute of Parasitology, Czech Republic

O. Alejandro Aleuy Young, Florida Atlantic University, United States

Sadaqat Ali, Islamia University of Bahawalpur, Pakistan

Leucio Alves, Federal Rural University of Pernambuco (UFRPE), Brazil

Caroline Amoroso, University of Virginia, United States

Peter Andrus, Shanghai Jiao Tong University School of Medicine, China

Mercy Ashepet, Royal Museum for Central Africa, Belgium

Jordana Assis, Universidade Federal de Minas Gerais, Brazil

Stephen Atkinson, Oregon State University, United States

Mehmet Aykur, Ege University, Turkey

Laura Backus, California Polytechnic State University, United States

Davide Badano, University of Siena, Italy

Estefanía Bagnato, Laboratorio de Investigaciones en Biodiversidad y Evolución (LIEB), Facultad de Ciencias Naturales y Ciencias de la Salud, Universidad Nacional de la Patagonia San Juan Bosco, Argentina

Berit Bangoura, University of Georgia College of Veterinary Medicine, United States

Luiz Daniel de Barros, Federal University of Lavras, Brazil

Cesar C. Bassetto, UNESP, Brazil

Walter Basso, Institute of Parasitology, University of Bern, Switzerland

Kelly Bateman, Cefas, United Kingdom of Great Britain and Northern Ireland

Chiara Bazzocchi, University of Milan, Italy

Daniel Becker, Montana State University System, United States

Brianna R. Beechler, Oregon State University, United States

María Beltrame, CONICET, Argentina

Mourad Ben Said, University of Manouba, Tunisia

Michal Benovics, Masaryk University, Czech Republic

Rasa Bernotienė, Nature Research Centre, Lithuania

Martha Betson, University of Surrey, United Kingdom of Great Britain and Northern Ireland

Ian Beveridge, University of Melbourne Faculty of Veterinary and Agricultural Sciences, Australia

Winston Black, St Francis Xavier University, Canada

David Blair, James Cook University, Australia

Damer Blake, Royal Veterinary College, United Kingdom of Great Britain and Northern Ireland

Isabel Blasco-Costa, Museum d'histoire naturelle de la Ville de Geneve, Switzerland

Warren Booth, Virginia Tech, United States

Antonio Bosco, University of Naples Federico II School of Agricultural Science and Veterinary Medicine, Italy

Alan Bowman, University of Aberdeen, United Kingdom of Great Britain and Northern Ireland

Geoffrey Boxshall, Natural History Museum, United Kingdom of Great Britain and Northern Ireland

Charles Addoquaye Brown, University of Ghana College of Health Sciences, Ghana

Daniel Browning Jr, Pearl River Community College, United States

Stephen Bullard, Auburn University, United States

Dora Buonfrate, Ospedale Sacro Cuore Don Calabria, Italy

Ian F. Burgess, Insect Research & Development Limited, United Kingdom of Great Britain and Northern Ireland

Amaya Bustinduy, London School of Hygiene and Tropical Medicine Faculty of Infectious and Tropical Diseases, United Kingdom of Great Britain and Northern Ireland

Joanne Cable, Cardiff University, United Kingdom of Great Britain and Northern Ireland

Rafael Calero-Bernal, Complutense University of Madrid, Spain

Nichola Calvani, University of Sydney, Australia

Lauren Camp, University of Wyoming College of Agriculture Life Sciences and Natural Resources, United States

Melissa E. Carew, The University of Melbourne, Australia

Jane Carlton, Johns Hopkins University, United States

Aitor Casas-Sanchez, LSTM, United Kingdom of Great Britain and Northern Ireland

Serena Cavallero, Università degli Studi di Roma La Sapienza, Italy

Nathalie Charbonnel, Centre de Biologie et de Gestion des Populations, France

Qin Cheng, Centre de Biologie et de Gestion des Populations, INRA-CBGP, France

Giovanni Cilia, Research Centre for Agriculture and Environment (CREA-AA), Council for Agricultural Research and Agricultural Economics Analysis, Bologna, Italy, Italy

Lavinia Ciuca, University of Naples Federico II, Italy

Sylvester Coleman, Liverpool School of Tropical Medicine, United Kingdom of Great Britain and Northern Ireland

Yolanda Corripio-Miyar, Moredun Research Institute, United Kingdom of Great Britain and Northern Ireland

James Cotton, University of Glasgow College of Medical Veterinary and Life Sciences, United Kingdom of Great Britain and Northern Ireland

Gaoussou Coulibaly, Université Félix Houphouët-Boigny, Côte d'Ivoire

Robert Cowie, University of Hawaii, United States

Thomas Cribb, University of Queensland, Australia

Armando Cruz-Laufer, Hasselt University, Belgium

Richard Culleton, Ehime University, Japan

Lucas Cunningham, Liverpool School of Tropical Medicine, United Kingdom of Great Britain and Northern Ireland

Alex Córdoba-Aguilar, Universidad Nacional Autonoma de Mexico, Mexico

John Dalton, National University of Ireland Galway, Ireland

Gabriel De La Torre, Universidade Federal do Paraná, Brazil

Carolina De Marco Verissimo, University of Galway College of Science and Engineering, Ireland

Dakeishla M. Diaz-Morales, DePaul University, United States

Carl Dick, Western Kentucky University, United States

Filippo Maria Dini, University of Bologna, Italy

Olgica Djurkovic-Djakovic, Institute for Medical Research, University of Belgrade, Serbia

Sheila Donnelly, University of Galway, Ireland

Stephen Doyle, Wellcome Sanger Institute, United Kingdom of Great Britain and Northern Ireland

Alison B. Duncan, Universite de Montpellier, France

Jenny Dunn, Keele University, United Kingdom of Great Britain and Northern Ireland

Jean Dupouy-Camet Dupouy-Camet, Universite Paris Cite Faculte de Sante, France

Haley Dutton, Auburn University, United States

John Ellis, University of Technology Sydney, Australia

Kathryn Else, University of Manchester, United Kingdom of Great Britain and Northern Ireland

Kristin Elwin, Public Health Wales Microbiology ABM, United Kingdom of Great Britain and Northern Ireland

Ananias Escalante, Arizona State University, United States

Claudia Esquivel, University of the Balearic Islands, Spain

Anna Faltýnková, Biology Centre Czech Academy of Sciences Institute of Parasitology, Czech Republic

María Cecilia Fantozzi, Universidad de Valencia, Spain

Majid Fasihi Harandi, Kerman University of Medical Sciences, Iran (the Islamic Republic of)

Miguel Fernandez-Garcia, Universidad CEU San Pablo, Spain

Marco Festa-Bianchet, Universite de Sherbrooke, Canada

Marian Flis, University of Life Sciences in Lublin, Poland

Hans-Peter Fuehrer, Veterinarmedizinische Universitat Wien, Austria

Martín Fugassa, CONICET Mar del Plata, Argentina

Bruno Fusaro, Instituto Antartico Argentino, Argentina

Shawn Gale, Brigham Young University, United States

Terry Galloway, University of Manitoba, Canada

Juan Garcia, Instituto Nacional de Tecnología Agropecuaria, Argentina

Luz Garcia-Longoria Batanete, University of Extremadura, Spain

Scott Gardner, University of Nebraska-Lincoln, United States

Miriam Garner, Moredun Research Institute, United Kingdom of Great Britain and Northern Ireland

Diego Garzon-Ospina, Universidad Pedagogica y Tecnologica de Colombia (UPTC), Colombia

Robin Gasser, The University of Melbourne, Australia

Timothy Geary, McGill University, Canada

Simona Georgieva, Universitat de Valencia Instituto Cavanilles de Biodiversidad y Biologia Evolutiva, Spain

David Gibson, The Natural History Museum, United Kingdom of Great Britain and Northern Ireland

Carmen Gilardoni, Laboratorio de Parasitología (LAPA), Instituto de Biología de Organismos Marinos (IBIOMAR, CCT CONICET-CENPAT), Argentina

Pawel Gladysz, Medical University of Gdańsk, Poland

Geoffrey Gobert, Queen's University Belfast, United Kingdom of Great Britain and Northern Ireland

Luis Gomez-Puerta, Universidad Nacional Mayor de San Marcos, Peru

Cynthya González, CONICET, Argentina

Roberto González Garduño, Universidad Autónoma Chapingo, Mexico

Catherine Gordon, QIMR Berghofer Medical Research Institute, Australia

Mónica Gozalbo, Universidad de Valencia, Spain

Daniel Grabner, Universitat Duisburg-Essen, Germany

Carlos Graeff-Teixeira, Universidade Federal do Espírito Santo, Brazil

Jaco M. Greeff, University of Pretoria, South Africa

Valentin Greigert, University of Reims Champagne-Ardenne, France

Chasen Griffin, University of Hawai'i at Manoa, United States

Alessandra Guarneri, Oswaldo Cruz Foundation Rene Rachou Institute, Brazil

Ricardo Guerrero, Universidad Central de Venezuela, Venezuela, Bolivarian Republic of

Dirceu Guilherme de Souza Ramos, Universidade Federal de Jataí, Brazil

Daniel Gustafsson, Guangdong Academy of Sciences Institute of Zoology, China

Bruce Halliday, Australian National Insect Collection, Australia

Omar Hamarsheh, Al Quds University Faculty of Science and Technology, Palestine, State of

Adam Hayward, Moredun Research Institute, United Kingdom of Great Britain and Northern Ireland

Shannon Hedtke, La Trobe University, Australia

Cyril Henard, University of Copenhagen, Denmark

Analía Henríquez, Universidad San Sebastián, Chile

Geoff Hide, Salford University, United Kingdom of Great Britain and Northern Ireland

Thomas Hiller, University of Ulm, Germany

Robert Hirt, Newcastle University, United Kingdom of Great Britain and Northern Ireland

John Hnida, Midwestern University, United States

Celia Holland, University of Dublin, Trinity College, Ireland

Astrid Holzer, Biology Center of the Academy of Sciences of the Czech Republic, Czech Republic

Jong Ha Hong, Centre for Eurasian Bioarchaeological Research, Institute of Korean Archaeology and Ancient History, Kyung Hee University, Seoul, Korea, Korea (the Republic of)

Dong Hoon Shin, Seoul National University College of Medicine, Korea (the Republic of)

Herve Hoste, INRAE, France

Michael H Hsieh, The George Washington University, United States

Xinyi Hua, University of Kentucky, United States

Daniel Huston, Commonwealth Scientific and Industrial Research Organisation, Australia

José Iannacone, Universidad Nacional Federico Villarreal, Peru

Angela Ionică, Universitatea de Stiinte Agricole si Medicina Veterinara din Cluj-Napoca, Romania

Furhan Iqbal, Bahauddin Zakariya University, Pakistan

Manuel Irigoitia, Universidad Nacional de Mar del Plata, Argentina

Joseph Jackson, University of Salford, United Kingdom of Great Britain and Northern Ireland

Daniel Jeffares, University of York, United Kingdom of Great Britain and Northern Ireland

Pablo Jimenez Castro, Antech Diagnostics Inc, United States

Agustín Jiménez Ruiz, Southern Illinois University Carbondale, United States

Lori Jones, Carleton University, Canada

Alexandra Juhasz, Liverpool School of Tropical Medicine, United Kingdom of Great Britain and Northern Ireland

Kerstin Junker, Agricultural Research Council, South Africa

Kumas Kaan, University of Copenhagen Faculty of Health and Medical Sciences, Denmark

Morakot Kaewthamasorn, Chulalongkorn University, Thailand

Panagiotis Karanis, University of Cologne, United States

Egil Karlsbakk, University of Bergen, Norway

Frank Katzer, Moredun Research Institute, United Kingdom of Great Britain and Northern Ireland

Irina Kaygorodova, FSBSI Limnological Institute of Siberian Branch of the Russian Academy of Sciences, Russian Federation

Jason Keegan, Trinity College Dublin, Ireland

Samuel Kelava, Hokkaido University, Japan

Jane Kelley, Australia Department of Primary Industries and Energy, Australia

Francis Kemngo, Texas Biomedical Research Institute, United States

Rua Khogali, ICIPE, Kenya

Alexander A. Kirillov, Russian Academy of Sciences, Russian Federation

Agnieszka Kloch, Polish Academy of Sciences, Poland

Sarah Knowles, University of Oxford, United Kingdom of Great Britain and Northern Ireland

Judith Kochmann, Johannes Gutenberg University Mainz, Germany

Alain Kohl, LSTM, United Kingdom of Great Britain and Northern Ireland

Iva Kolarova, Charles University Faculty of Science, Czech Republic

Jennifer Koop Koop, Northern Illinois University, United States

Andrew C. Kotze, University of Queensland, Australia

Laura Kramer, University of Parma, Italy

Boris Krasnov, Jacob Blaustein Institutes for Desert Research, Ben-Gurion University of the Negev, Israel

Delane Kritsky, idaho state university, United States

Roman Kuchta, Biology Centre Czech Academy of Sciences Institute of Parasitology, Czech Republic

Rajender Kumar, ICAR-National Research Centre on Equines, India

Yuriy Kuzmin, I I Schmalhausen Institute of Zoology National Academy of Sciences of Ukraine, Ukraine

E. James La Course, Liverpool School of Tropical Medicine, United Kingdom of Great Britain and Northern Ireland

Roz Laing, University of Glasgow, School of Veterinary Medicine, United Kingdom of Great Britain and Northern Ireland

Olimpia Lamberti, London School of Hygiene & Tropical Medicine, United Kingdom of Great Britain and Northern Ireland

Fernando Lares-Villa, Instituto Tecnológico de Sonora, Mexico

Maria Latrofa, University of Bari, Italy

Matthieu Le Bailly, University of Bourgogne Franche-Comte, France

Marissa Ledger, McMaster University, Canada

Alex Lees, Manchester Metropolitan University, United Kingdom of Great Britain and Northern Ireland

Tommy Leung, University of New England, Australia

Kun Li, Nanjing Agricultural University, China

Lisette van Lieshout, Leiden University Medical Center, Netherlands

Kevin Lievano, University of Nebraska-Lincoln, United States

Jessica Light, Texas A&M Univ, United States

Catherine Lilley, University of Leeds, United Kingdom of Great Britain and Northern Ireland

Andrés Link, Universidad de los Andes, Colombia

Sam Loker, University of New Mexico, United States

Elaine Lopes Carvalho, Federal Rural University of Amazonia, Brazil

Welmoed van Loon, Free University of Berlin, Germany

Lien Luong, University of Alberta, Canada

Julio López-Abán, Universidad de Salamanca Facultad de Farmacia, Spain

Ramón López-Gijón, Universidad de Granada Facultad de Medicina, Spain

Jean-Francois Magnaval, Toulouse University Hospitals, France

Arnaldo Maldonado, Fundação Oswaldo Cruz, Brazil

Paulo Mangini, Universidade Federal do Paraná, Brazil

Valentina Marchese, University of Turin, Italy

Richard Martin, Iowa State University, United States

Chris Marufu, University of Pretoria Faculty of Veterinary Science, South Africa

Virginia Marugan-Hernandez, Royal Veterinary College, United Kingdom of Great Britain and Northern Ireland

Makia Masong, Catholic University of Central Africa, Cameroon

Maria Paola Maurelli, University of Naples Federico II, Italy

Isabel Mauricio, Instituto de Higiene e Medicina Tropical, Portugal

Clifton McKee, Johns Hopkins Bloomberg School of Public Health, United States

Sergei Medvedev, Zoologiceskij institut RAN, Russian Federation

Jairo Alfonso Mendoza-Roldan, University of Bari, Valenzano, Italy, Italy

Santiago Merino, Museo Nacional de Ciencias Naturales, Spain

Tara Merrill, Cary Institute of Ecosystem Studies, United States

Adriano Minichino, University of Naples Federico II, Italy

Mark A. Mitchell, Louisiana State University, United States

Martina Miterpaková, Parasitological Institute of the Slovak Academy of Sciences, Slovakia

Xavier de Montaudouin, University of Bordeaux, France

German Augusto Murrieta Morey, Universidad Nacional de la Amazonia Peruana, Peru

Lawrence Mugisha, Makerere University, Uganda

Micky M. Mwamuye, Africa Nazarene University, Kenya

Kioko Mwikali, KEMRI-Wellcome Trust Research Programme, Kenya

Elmarie Myburgh, Hull York Medical School, United Kingdom of Great Britain and Northern Ireland

Maria Müller, UNIFESP, Brazil

Rodrigo Narciso, São Paulo State University, Brazil

Kok-Boon Neoh, National Chung Hsing University, Taiwan

Edward Netherlands, University of the Free State, South Africa

Kristina Noreikiene, Vilnius University, Lithuania

Cormac J. O'Shea, Technological University of the Shannon, Ireland

Benjamin Ofori, University of Ghana, Ghana

Akinola Oluwole, Sightsavers, Nigeria

Naidu Ommi, University of Hyderabad, India

Luis Miguel Ortega-Mora, Complutense University of Madrid, Spain

Guadalupe Ortega-Pierres, Centro de Investigación y de Estudios Avanzados, Mexico

Mamadou Ouattara, Universite Felix Houphouet-Boigny, Côte d'Ivoire

Pinar Ozdemir, Sisli Hamidiye Etfal Egitim ve Arastirma Hastanesi Deri ve Zuhrevi Hastaliklar Klinigi, Turkey

Camila Pantoja, 2Curso de Pós-Graduação em Ciências Veterinárias, Brazil

Lais Pardini, Facultad de Cs Veterinarias, Argentina

Fabiano Paschoal, Universidade do Estado do Rio de Janeiro, Brazil

Rachel Penczykowski, Washington University in St Louis, United States

Andy Peters, University of Edinburgh, United Kingdom of Great Britain and Northern Ireland

Khanh Phama, Weill Cornell Medicine, United States

Wojciech Piasecki, University of Szczecin, Poland

David Pickel, University of Warsaw, Poland

Hudson Pinto, Universidade Federal de Minas Gerais, Brazil

Robert Poulin, University of Otago, New Zealand

Rosemonde Power, Stockholm University, Sweden

Katharine Preedy, James Hutton Institute, United Kingdom of Great Britain and Northern Ireland

Bronwen Presswell, University of Otago, New Zealand

Iva Přikrylová, University of Limpopo - Turfloop Campus, South Africa

Rupert Quinnell, Leeds University, United Kingdom of Great Britain and Northern Ireland

Reem Ramadan, Cairo University, Egypt

Darío Ramirez, CONICET Cordoba, Argentina

Lisa Ranford-Cartwright, University of Glasgow, United Kingdom of Great Britain and Northern Ireland

Benjamin Reilly, Carnegie Mellon University - Qatar, Qatar

Florian Reyda, State University of New York College at Oneonta, United States

Lisa Reynolds, University of Victoria, Canada

Mark Robinson, Queen's University Belfast, United Kingdom of Great Britain and Northern Ireland

Sónia Rocha, University of Porto Institute of Biomedical Sciences Abel Salazar, Portugal

Alicia Rojas, University of Costa Rica | UCR Centro de Investigación en Enfermedades Tropicales, Costa Rica

David Rollinson, The Natural History Museum, United Kingdom of Great Britain and Northern Ireland

Silvia Roure, Hospital Germans Trias i Pujol, Spain

Andre V. Rubio, Universidad de Chile, Chile

Roberto Sacchi, Università degli Studi di Pavia, Italy

Maria Santos, University of Porto Faculty of Sciences, Portugal

Bahador Sarkari, Shiraz University of Medical Sciences, Iran (the Islamic Republic of)

Abhay Satoskar, Ohio State University, United States

Salvatore Scarso, IRCCS Ospedale Sacro Cuore Don Calabria, Italy

Tomas Scholz, Institute of Parasitology, AS CR, Czech Republic

W. Evan Secor, Centers for Disease Control and Prevention, United States

Mauricio Seguel, University of Guelph, Canada

Fabiano Sellos Costa, Federal Rural University of Pernambuco, Brazil

Emily Severance, Johns Hopkins School of Medicine, United States

Shookofeh Shamsi, Charles Sturt University, Faculty of Science, Wagga Wagga, Australia

Elliot Keats Shwab, Duke University Medical Center, United States

Aline Silva, National Institutes of Health, United States

Raquel Simões, Universidade Federal Rural do Rio de Janeiro, Brazil

Shanker Singh, College of Veterinary Science and Animal Husbandry, DUVASU, India

Abhinav Sinha, National Institute of Malaria Research, India

Padet Siriyasatien, Chulalongkorn University, Thailand

Jan Slapeta, University of Sydney, Australia

Sergey Slepchenko, Institute of Problems of Northern Development of the Tyumen Scientific Centre of Siberian Branch of the Russian Academy of Sciences, Russian Federation

Nico Smit, North-West University, South Africa

Adrian Smith, University of Oxford, United Kingdom of Great Britain and Northern Ireland

Brenda Solorzano-Garcia, Universidad Nacional Autonoma de Mexico, Mexico

Sripoorna Somasundaram, The Ohio State University, United States

Aneta Spyra, University of Silesia, Poland

Michal Stanko, Slovak Academy of Sciences, Slovakia

J. Stothard, Liverpool School of Tropical Medicine, United Kingdom of Great Britain and Northern Ireland

Adrian Streit, Max Planck Institute for Developmental Biology, Germany

Amy Sturt, Stanford University, United States

Chunlei Su, The University of Tennessee,, United States

Keisuke Suganuma, Obihiro University of Agriculture and Veterinary Medicine, Japan

Bernd Sures, University of Duisburg-Essen, Germany

Colin Sutherland, London School of Hygiene and Tropical Medicne, United Kingdom of Great Britain and Northern Ireland

Yaroslav Syrota, Slovak Academy of Sciences, Slovakia

Csaba Székely, Hungarian Academy of Sciences Institute for Veterinary Medical Research, Hungary

Pavel Široký, University of Veterinary and Pharmaceutical Sciences, Faculty of Veterinary Hygiene and Ecology, Czech Republic

Ala Tabor, The University of Queensland, Australia

Weerapol Taweenan, Khon Kaen University, Thailand

Claire Tietelbaum, University of Georgia Warnell School of Forestry & Natural Resources, United States

Juan Timi, Universidad Nacional de Mar del Plata Facultad de Ciencias Exactas y Naturales, Argentina

Ilaria Trave, University of Genoa, Italy

Eva Tyden, Sveriges lantbruksuniversitet, Sweden

Gérald Umhang, Anses Rabies and Wildlife Laboratory, France

Aleksandra Uzelac, Institute for Medical Research, Serbia

Letícia Úngari, Universidade Federal de Uberlândia Instituto de Ciências Biomédicas, Brazil

Robin Varney, William & Mary Virginia Institute of Marine Science, United States

Luigi Venco, University of Torino Library System, Italy

José M. Venzal, Universidad de la Republica Uruguay Facultad de Veterinaria, Uruguay

Lonneke Vervelde, Gezondheidsdienst voor Dieren BV, Netherlands

Jaco Verweij, Elisabeth-TweeSteden Ziekenhuis, Netherlands

Thiago Bernardi Vieira, Universidade Federal do Para, Bahamas

Maxim Vinarski, Omsk State Pedagogical University, Russian Federation

Hannah Vineer, University of Liverpool Faculty of Science, United Kingdom of Great Britain and Northern Ireland

Mark Viney, University of Bristol, United Kingdom of Great Britain and Northern Ireland

Fernanda Silveira Flores Vogel, Universidade Federal de Santa Maria (UFSM), Brazil

Petr Volf, Charles University, Czech Republic

Jan Votypka, Charles University, Czech Republic, Czech Republic

Runxi Wang, The University of Hong Kong, Hong Kong

Tianyi Wang, Jilin University, China

Lesley Warner, South Australian Museum, Australia

Peter Wasson, LifeArc, United Kingdom of Great Britain and Northern Ireland

Denise Wawman, University of Oxford, United Kingdom of Great Britain and Northern Ireland

Bonnie Webster, Natural History Museum, United Kingdom of Great Britain and Northern Ireland

Sarah Wheeler, Sacramento-Yolo Mosquito and Vector Control District, United States

Andrew Williams, University of Copenhagen, Denmark

Kathryn H. Wiltshire, Primary Industries and Regions of South Australia, Australia

Kerry Woods, University of Bern, Switzerland

Hiroshi Yamasaki, National Institute of Infectious Diseases, Japan

Hui-Yuan Yeh, Nanyang Technological University, Singapore

Helene Yera, Université de Limoges, France

Jinyong Zhang, Chinese Academy of Sciences, China

Francisco Zaragoza Tapia, Universidad Autonoma del Estado de Hidalgo, Mexico

Qun Zhang, Wuhan University, China

Jinlin Zhou, Chinese Academy of Agricultural Sciences Shanghai Veterinary Research Institute, China

Shelby Ziegler, Villanova University, United States

Annetta Zintl, University College Dublin, Ireland
